# Optimizing Chronotherapy in Psychiatric Care: The Impact of Circadian Rhythms on Medication Timing and Efficacy

**DOI:** 10.3390/clockssleep6040043

**Published:** 2024-11-05

**Authors:** Cezar-Ivan Colita, Dirk M. Hermann, Madalina Filfan, Daniela Colita, Thorsten R. Doepnner, Oana Tica, Daniela Glavan, Aurel Popa-Wagner

**Affiliations:** 1 Doctoral School, University of Medicine and Pharmacy Carol Davila, 050474 Bucharest, Romania; cezar.ivan23@gmail.com (C.-I.C.); danielacolita@gmail.com (D.C.); 2 Department of Neurology, University Hospital Essen, University of Duisburg-Essen, 45147 Essen, Germany; dirk.hermann@uk-essen.de; 3 Department of Psychiatry, University of Medicine and Pharmacy, 200349 Craiova, Romania; filfanmadalina@gmail.com; 4 Department of Neurology, University Medical Center, Klinikstraße 33, 35392 Gießen, Germany; thorsten.doeppner@neuro.med.uni-giessen.de; 5 Department of Pharmacology, University of Medicine and Pharmacy, 200349 Craiova, Romania; oanasorinatica@gmail.com

**Keywords:** circadian rhythmicity, cortisol, melatonin, serotonin, dopamine, clock genes, chronopharmacology

## Abstract

In many medical settings, medications are typically administered in the morning or evening, aligning with patients’ daily routines. This practice does not stem from chronotherapy, which involves scheduling drug administration to enhance its effectiveness, but rather from the way clinical operations are structured. The timing of drug administration can significantly affect a medication’s effectiveness and side effects, with the impact varying by up to ten times based on circadian rhythms. Disorders such as major depression, bipolar disorder, and schizophrenia are linked to disruptions in these rhythms. Recent studies have found that circadian dysfunctions, including genetic and neurohumoral changes, underlie many psychiatric conditions. Issues such as an altered glucocorticoid rhythm due to impaired HPA axis function, disturbed melatonin balance, and sleep disturbances have been noted in psychotic disorders. Furthermore, mood disorders have been associated with changes in the expression of circadian rhythm genes such as *Clock*, *Bmal1*, and *Per*. Considering that the absorption, biodistribution, effects on target organs, half-life, metabolism, and elimination of drugs are all influenced by the body’s circadian rhythms, this narrative review explores the optimal timing of medication administration to maximize efficacy and minimize side effects in the treatment of psychiatric disorders. By closely monitoring circadian variations in cortisol, melatonin, and key clock genes, as well as by deepening our understanding of the metabolisms and pharmacokinetics of antipsychotic medications, we propose a chronotherapy approach for psychiatric patients that could significantly enhance patient care.

## 1. Introduction

The physiological processes of our body, such as the sleep–wake cycle, gene transcription, metabolic activities, body temperature, blood pressure, immune system responses, and neuroendocrine and behavioral changes, adhere to a pattern regulated by our internal clock. This internal clock, in turn, synchronizes with environmental cues like the day–night sequence governed by the sun and orchestrates a wide range of molecular, physiological, and behavioral processes. Consequently, it is unsurprising that disturbances in this internal clock are linked to various health conditions, including heart disease, diabetes, obesity, cancer, and psychiatric disorders [1,2].

### Brain Circuits That Control the Internal Clock

In humans, the suprachiasmatic nuclei (SCN) in the hypothalamus act as our circadian pacemaker and are synchronized to the 24 h solar day cycle via the retinohypothalamic tract (RHT) and the geniculohypothalamic tract (GHT). These tracts connect the retina and SCN through the intergeniculate leaflet (IGL) in the thalamus, aligning our internal biological rhythms with the external environment. Notably, when the SCN is isolated in organotypic culture, its independent timing mechanism can persist indefinitely with remarkable precision and robustness. This persistence suggests significant evolutionary conservation of circadian regulation mechanisms [3].

**Figure 1:**
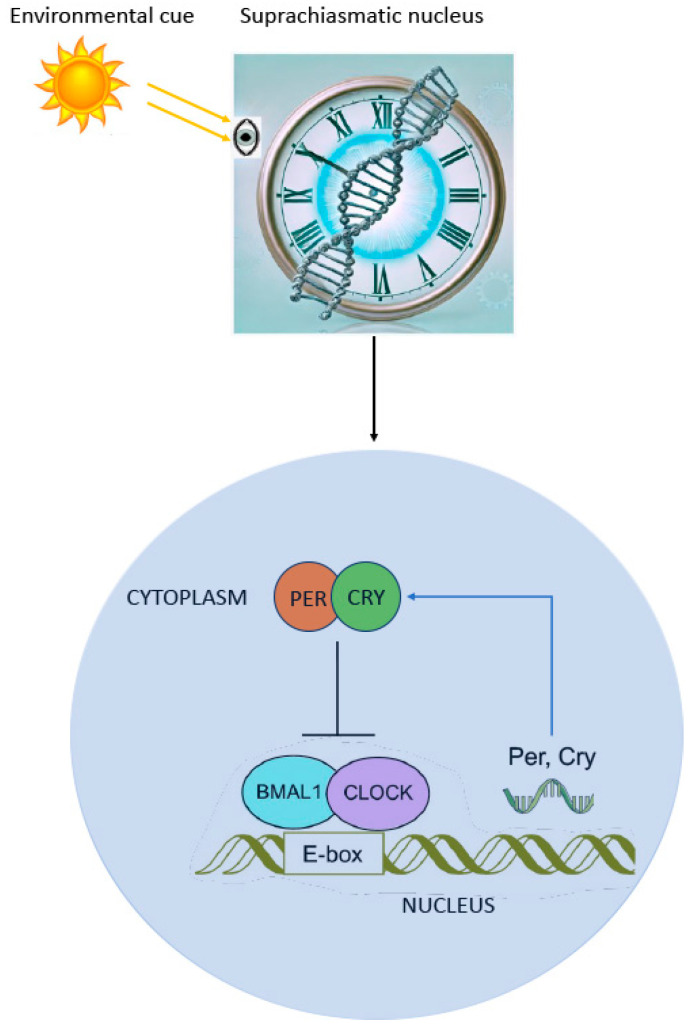
The suprachiasmatic nuclei (SCN) in the hypothalamus serve as the circadian pacemaker, synchronized to the 24 h day via the retinohypothalamic and geniculohypothalamic tracts, which link the retina and SCN. The circadian rhythm is regulated by core clock genes, with the *Clock* gene playing a key role. The CLOCK protein forms a heterodimer with BMALl, binding to E-box elements on DNA to activate the Period (*Per*) and Cryptochrome (*Cry*) genes, thereby driving the rhythmic regulation of biological processes.

The circadian rhythm within the SCN nucleus is regulated by a complex of genes, likely a consequence of the increasing complexity of organisms benefiting from a circadian oscillator. In humans, one of the most crucial genes in the endogenous master clock system is the Circadian Locomotor Output Cycles Kaput (*Clock*) gene. Its primary function involves activating downstream core clock genes through transcription and promoting rhythmic chromatin opening, thereby regulating the accessibility of other transcription factors to DNA. In brief, to maintain the core circadian rhythm, the CLOCK protein can form a heterodimer with BMAL1 (brain and muscle Arnt-like protein-1). This heterodimer binds to E-box enhancer elements upstream of Period (*Per1*, *Per2*, *Per3*) and Cryptochrome (*Cry1*, *Cry2*) genes, thereby activating their transcription (Figure 1).

However, recent work has shown that the Neuronal PAS Domain Protein 2 (NPAS2) can compensate for the loss of *Clock1* in peripheral cells and in the SCN, also suggesting that the *Clock1* gene is not the most critical [4].

Impairments in the rhythmic oscillation of several clock genes seem to be critical for adaptive behavior and normal body physiology. Indeed, forced expression of *Per1* and *Per2* in animal models has been associated with impairments in the behavioral and circadian rhythms of other clock genes [5]. Furthermore, constitutive, non-rhythmic expression has been reported for *Clock1* in peripheral white blood cells obtained from patients with coronary heart disease [6] and in the prefrontal cortex of aged postmortem human brains [7]. Therefore, it is essential for the organism as a whole that the various clocks in individual tissues are synchronized by the central clock, thereby allowing the system to function more effectively [8].

Furthermore, the activity of the SCN is influenced by non-light-related information, including neurotransmitters like serotonin [9,10] and melatonin [11] and inputs from internal clocks in peripheral tissues [12].

Signals from serotonin produced in the raphe nuclei are important for modulating SCN responses to non-photic cues [13]. Additionally, various non-photic factors, including food availability, social interactions, and exercise, can influence the circadian clock or adjust the circadian phase of external oscillators [14].

Light indirectly influences serotonin synthesis. During daylight, increased light exposure can enhance serotonin production and release by the raphe nucleus. Conversely, decreased light (darkness) reduces this activity. The SCN regulates the pineal gland’s production of melatonin through a multi-step pathway involving the spinal cord and the superior cervical ganglia (SCG). In the absence of light, the pineal gland is stimulated to produce melatonin by first converting tryptophan into serotonin and then serotonin into melatonin [15]. Melatonin secreted by the pineal gland feeds back to the SCN to modulate body circadian rhythms, effectively signaling the body to prepare for sleep during night-time.

At night, complex networks of efferent neurons from the SCN extend to the pineal gland, where they release norepinephrine. This action activates beta-1 and alpha-1 adrenergic receptors on pinealocytes, initiating the conversion of serotonin, produced during daylight in the pineal gland, into melatonin [16]. However, there is no proof that serotonin from the raphe nuclei stops the pineal gland from producing melatonin. Recent studies have revealed that the timing of circadian rhythms leads to varied expression in numerous genes not directly linked to circadian rhythms. These genes primarily play roles in autonomic, homeostatic, and emotional control in rodents [17]. In contrast, the circadian patterns observed in Syrian hamsters closely resemble those in humans, unlike the patterns observed in rats. Consequently, we should not directly apply observations concerning circadian fluctuations in different hormones found in mice or rats, which are nocturnal animals, to humans, who are diurnal [18].

## 2. Circadian Rhythmicity and Psychiatric Disorders

Four key characteristics of disrupted circadian rhythms are thought to play a role in the emergence of mood disorders: (1) a misalignment with the 24 h environmental cycles, (2) a lack of harmony between the body’s internal clocks, (3) diminished strength of biological rhythms, and (4) alterations in sleep pattern [19].

### 2.1. Clock Genes and Mental Health

Studies focusing on specific genes and their expression have indicated a significant role for the *CLOCK* gene in the development of schizophrenia and in the circadian rhythm abnormalities observed as side effects of treatment [20,21,22]. Furthermore, research into human genetics and gene expression has revealed a connection between *CLOCK* gene activity and substance abuse. The impact of substance abuse on *CLOCK* gene expression seems quite evident [23,24]. Conversely, changes in circadian rhythms, specifically in the wake–sleep cycle caused by antipsychotic medications, provide a valuable method to evaluate the effectiveness of chronotherapies in treating psychiatric conditions.

Previous research has shown that mutations in the clock genes, specifically the timeless homolog gene (*TIMELESS*) and the period homolog 3 gene (*PER3*), are linked to mental health conditions such as schizophrenia and bipolar disorder [21,22]. Similarly, research analyzing single-nucleotide polymorphisms (SNPs) has associated the *CLOCK* gene with bipolar disorders. Indeed, the examination of SNPs across 19 circadian rhythm genes (including *BMAL1*, *BHLHB2*, *BHLHB3*, *CLOCK*, *CRY1*, *CRY2*, *CSNK1E*, *DBP*, *NPAS2*, *NR1D1*, *PER1*, *PER2*, *PER3*, *RORA*, *TIMELESS*, *VIP*, and *VIPR2*) revealed that the *CRY1* and Neuronal PAS Domain Protein 2 (*NPAS2*) genes are linked to unipolar major depression, while the *CLOCK* and *VIP* genes are associated with bipolar disorder [25].

Nevertheless, the link between genetics and psychiatric disorders has yet to be conclusively established, largely due to the limited number of participants in studies. Despite this, the clinical significance of these findings continues to be contested. Many clinical studies have not found a direct link between the SNPs in the *CLOCK* gene and the occurrence or symptoms of mood disorders, leading to inconsistent results. This discrepancy may be the result of studies not accounting for gender differences or not targeting specific demographic groups. Furthermore, although the genetic basis of schizophrenia is still not fully understood, focused research on certain genes and their expression levels has hinted at the involvement of the *CLOCK* gene in both the development of schizophrenia and the regulation of circadian rhythm disturbances that often accompany the disorder [21,22].

However, there is a bidirectional relationship between *CLOCK* gene expression and drug abuse. Genetic predispositions, reflected in variations in the *CLOCK* gene, can influence a patient’s risk of drug abuse. Conversely, drug abuse can disrupt the normal expression of the *CLOCK* gene, leading to further dysregulation of circadian rhythms and potentially worsening addictive behaviors. This complex interaction underscores the importance of considering genetic factors in the prevention and treatment of substance abuse disorders [22,23,24].

A recent study, which included 413 patients with mood disorders and compared them to 1294 patients without these conditions, investigated the relationship between mood disorders and various clock genes, such as *BHLHB2*, *CLOCK*, *CSNK1E*, *NR1D1*, *PER2*, *PER3*, and *TIMELESS*, through allele, genotype, and haplotype analyses. Furthermore, the study employed the Multifactor Dimensionality Reduction (MDR) method, a non-parametric and model-free approach, to assess gene–gene interactions. The findings highlighted significant associations between mood disorders and specific alleles, notably *TIMELESS* rs4630333 and *CSNK1E* rs135745, in relation to major depressive disorder and bipolar disorder, as well as a notable association with a *CLOCK* gene haplotype. These findings underscore the critical roles of clock genes and their interactions within the circadian systems in the development of mood disorders [26].

**Figure 2:**
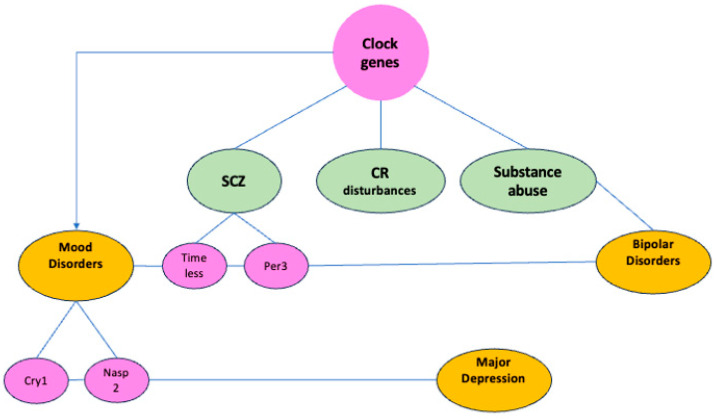
The diagram shows how different genes, such as the CLOCK gene, TIMELESS, PER3, CRY1, and NPAS2, are connected to mental health conditions like schizophrenia, bipolar disorder, and unipolar major depression. It also highlights the study on mood disorders illustrating their relevance to the circadian rhythm and mental health. Abbreviations: SCZ, schizophrenia; CR, circadian rhythm.

Another study conducted a detailed examination of time-of-death analysis through gene expression data from high-quality postmortem brains. It focused on 24 h cyclic patterns in six cortical and limbic areas of 55 patients without any psychiatric or neurological disorders (referred to as “controls”) and 34 patients diagnosed with major depressive disorder (MDD). In each examined region, several hundred transcripts exhibited 24 h cyclic patterns in the control group, with more than 100 transcripts showing consistent rhythmicity and phase alignment across regions. Key rhythmic genes included well-known clock genes such as *BMAL1*, *PER1*, *PER2*, *PER3*, *NR1D1*, *DBP*, *BHLHE40 (DEC1)*, and *BHLHE41 (DEC2)*. However, these cyclic patterns were significantly diminished in the brains of MDD patients, characterized by altered peak times and potentially disrupted phase connections among several circadian genes. This comprehensive analysis reveals a rhythmic oscillation of gene expression in areas beyond the SCN nucleus in subjects without disorders [27]. A graphical representation of the relationships between clock genes and mental health is shown in Figure 2.

### 2.2. Modulators of the Circadian Rhythmicity

#### 2.2.1. Melatonin and Cortisol

Mood disorders are often associated with disruptions in circadian clock-controlled functions, such as sleep patterns and cortisol secretion. Conversely, the disturbance of circadian rhythms—whether due to experiences like jet lag, night-shift work, or exposure to artificial light at night—has the potential to initiate or exacerbate affective symptoms in susceptible individuals [28].

Melatonin serves as a signal, transmitting information about the duration of daylight and darkness throughout the year to every cell, including those in the SCN nucleus (SCN). This highlights the variations in day–night cycles with the seasons, emphasizing melatonin’s role in enabling adaptation to these changes [29,30].

Similarly, fluctuations in adrenal steroid hormone levels, which vary with the day–night cycle, have been implicated in the onset of insomnia and other psychiatric disorders [31,32]. The release of cortisol is significantly influenced by the activity of the hypothalamus–pituitary–adrenal gland axis (HPA). This axis is regulated by the central pacemaker, which governs the circadian timing of corticotropin-releasing hormone (CRH) production in the paraventricular nucleus. This production is further enhanced by physical and emotional stressors. CRH then prompts the secretion of the adrenocorticotropic hormone (ACTH) from the corticotropin cells of the anterior pituitary gland, leading to the release of glucocorticoid cortisol from the adrenal cortex. In response, cortisol applies inhibitory feedback at both the pituitary and hypothalamus levels, forming a classic negative feedback loop. However, this feedback mechanism does not extend to the SCN [33].

#### 2.2.2. Dopamine and Serotonin

Dopamine is produced by small clusters of neurons in the mesencephalon (specifically, the ventral tegmental area and the substantia nigra), as well as in the diencephalon. In these regions, dopamine not only influences but is also influenced by the genetic mechanisms that control biological clocks, suggesting its crucial role in maintaining the rhythmic functioning of these brain areas [34]. For instance, both mammalian and fish retinas experience a daily fluctuation in dopamine levels, with these concentrations peaking during the day and declining at night. However, this cycle of dopamine in the retina appears to be influenced by melatonin, a hormone that promotes sleep in dark conditions [35].

Recent research on behavior and molecular biology underscores the *Clock* gene’s pivotal role in neural functions, especially in regulating dopaminergic pathways critical to understanding various psychiatric disorders. Evidence supporting the circadian control of the dopamine system includes research on canonical clock proteins that directly influence dopamine expression and regulation. One notable example is REV-ERBalpha (nuclear receptor subfamily 1, group D, member 1, NR1D1), a circadian nuclear receptor that negatively regulates the TTFL by targeting *Bmal1* mRNA [36]. This has been shown to affect dopamine production in the midbrain through the suppression of tyrosine hydroxylase (*Th*) mRNA synthesis and cholecystokinin (CCK), among others [36,37,38].

Interestingly, a recent comprehensive study on circadian gene expression in the primate brain questions the primacy of the hypothalamic nuclei as the master regulators of circadian physiology. Surprisingly, it was discovered that nuclei typically associated with rhythmic functions exhibit only mild-to-minimal rhythmic expression of transcripts. Instead, the prefrontal cortex exhibits the highest number of circadian cycling transcripts, followed by the medial globus pallidus, putamen, lateral globus pallidus, and mammillary bodies, which are part of the hypothalamic nuclei. Conversely, the SCN showed a very low number of cycling transcripts. Notably, this study demonstrated cyclic expression of dopamine D2 receptors in the putamen. Although the study does not prove the presence of intrinsic autonomous circadian clocks in the aforementioned brain areas with high cycling gene activity, this discovery may explain why drugs that block dopamine receptors, such as haloperidol, are effective as antipsychotic treatments [39].

The substantia nigra plays a pivotal role in the production of dopamine and is considered a crucial part of the basal ganglia network, acting as an initial gateway and significantly influencing its functionality. When the substantia nigra or its connected structures are damaged due to events like strokes or neurodegenerative conditions, a variety of neurological disorders can arise. According to Van Hooijdonk and colleagues, [40], such disorders encompass Parkinson’s disease, Huntington’s disease, Tourette syndrome, schizophrenia, ADHD, and OCD. Additionally, it is important to note that despite the lack of direct links from the nigrostriatal system to the SCN nuclei, the effect of dopamine on circadian rhythms is significant. This influence, which relates to both physiological and psychological disorders, appears to be mediated indirectly via serotonin pathways.

Dopamine receptors are found both in the central nervous system and in peripheral areas. Increasing evidence points to dopamine’s involvement in the regulation of circadian rhythms within these brain areas, either directly or indirectly, through multiple pathways and via different dopaminergic receptors located centrally and peripherally, highlighting its significant function.

## 3. Psychiatric Diseases and Circadian Physiology

Circadian disturbances are common in major depressive illness, bipolar disorder, and schizophrenia, as summarized by Boiko and colleagues [41]. These disruptions, which include changes in the sleep–wake cycle, core body temperature rhythms, and the secretion of melatonin and cortisol, are prevalent across almost all psychiatric conditions. The significance of these circadian abnormalities in the pathology of psychiatric disorders is currently under debate. Theories suggest that these disruptions may play a role in the onset and progression of mental illnesses in various ways: (1) as a primary factor, stemming directly from genetic predispositions in the circadian system that increase susceptibility to psychiatric conditions; (2) as a secondary factor, due to changes in the timing of behaviors associated with the illness, leading to a misalignment of rhythms; or (3) as a concurrent factor, due to shared molecular and neural pathways between psychiatric disorders and the circadian system [42].

### 3.1. Bipolar Disorders

Alterations in sleep patterns and architecture are key indicators of a depressive episode, observed in both bipolar disorder and major depressive disorder. An alternative perspective suggests that the erratic behaviors in patients with bipolar disorder could cause disturbances in their circadian rhythms, indicating a bidirectional interaction [43]. Fluctuations in daily melatonin levels, often showing increased levels during the day, have been associated with episodes of both bipolar disorder and major depression [44,45]. However, there is still no consensus among scientists on this issue. For instance, a significant decrease in melatonin levels was found in the cerebrospinal fluid (CSF) of patients with bipolar disorder, a finding not observed in patients with major depressive disorder. Conversely, a notable reduction in melatonin was detected in the blood serum of patients with major depressive disorder but not in those with bipolar disorder. Also, no correlation was found between serum and CSF melatonin levels or between melatonin levels and the severity of symptoms or sleep disturbances in either disorder [46].

At the molecular level, the analysis of saliva cortisol and cheek cell gene expression in patients revealed significant advancements in the expression profiles of the *Bmal1*, *Per1*, and *Nr1d1* genes during manic episodes compared to depressive episodes [47]. Patients exhibited a tendency towards advanced gene expression profiles during manic episodes when compared to controls but not during depressive episodes. The intensity of *Nr1d1* gene expression also varied between mania and depression [48]. After treatment, circadian phases resembled those of healthy individuals, suggesting that the expression profiles of certain clock genes could help to monitor the effectiveness of conventional treatments for bipolar disorders [47].

### 3.2. Schizophrenia

Various aspects of human behavior, including sleep patterns and cognitive abilities, as well as physiological traits such as body temperature, blood pressure, the onset of migraines, and hormone levels in the bloodstream, exhibit rhythms occurring every 12 h. In the case of blood pressure and body temperature, these 12 h rhythms act as a secondary layer to the more prominent 24 h cycle, suggesting the presence of multi-layered rhythmic patterns [49]. Thus, one study identified 12 h cycles of gene expression within the dorsolateral prefrontal cortex—a brain area crucial for cognitive functions—peaking during transitions between sleep and wakefulness (around 9 a.m./p.m.) or remaining constant (around 3 p.m./a.m.). Further, in the postmortem brains of patients diagnosed with schizophrenia, there is a disruption of these 12 h cycles in genes associated with protein folding and neuronal structural integrity [50].

Stress, and specifically the response of the HPA axis to it, is also recognized as a contributing factor in the development of schizophrenia and elevated cortisol levels, and it may serve as an indicator of this disorder [51,52].

## 4. Neural Pathways Involved in Psychiatric Diseases

The drugs currently used to treat psychotic patients primarily target the neurotransmitters dopamine and serotonin. This discovery stems from an investigation into medications that were originally found, by chance, to be effective for psychiatric conditions.

As a member of the catecholamine family of neuromodulators, dopamine significantly contributes to behaviors related to motivation, reward, and addiction [53]. It is essential for both eating and movement behaviors, positioning it as a potential key player in the entrainment of circadian activity rhythms through scheduled feeding or other rewarding stimuli [34,54,55,56].

First-generation antipsychotics, also known as typical antipsychotics, work by blocking dopamine receptors. This category includes drugs such as prochlorperazine, triflupromazine, haloperidol, chlorprothixene, loxapine, or pimozide. Among these, haloperidol, which has been in clinical use since 1958, is the most frequently prescribed medication in this group [57]. These medications are particularly effective at controlling the positive symptoms of schizophrenia, such as hallucinations and delusions, significantly reducing the likelihood of a subsequent psychotic episode.

Atypical antipsychotics, also known as second-generation antipsychotics, work by blocking serotonin and dopamine receptors. These medications are effective in managing both the positive and negative symptoms associated with schizophrenia, such as social withdrawal and ambivalence. Additionally, they have a lower risk of triggering a relapse [58].

Unlike traditional medications, these atypical variants affect the neurotransmission of serotonin (5-HT), norepinephrine, and/or histamine. This diverse mechanism of action is believed to contribute to their beneficial effects on mood and anxiety disorders, as outlined by Grinchii and Dremencov [59].

Recognized serotonin–dopamine antagonists include risperidone, olanzapine, quetiapine, ziprasidone, aripiprazole, paliperidone, asenapine, lurasidone, iloperidone, cariprazine, brexpiprazole, and clozapine, as noted by Haddad and colleagues [60]. Notably, certain atypical antipsychotics, like clozapine, olanzapine, and quetiapine, are known to interact with histamine-1 (H1) receptors [61]. The role of central histamine in regulating sleep, cognition, memory, and emotions has recently garnered interest for its therapeutic implications [62].

Clozapine, recognized for its efficacy in treating treatment-resistant schizophrenia, faced early reports of serious side effects such as agranulocytosis. This led to its temporary withdrawal and subsequent reintroduction with stringent monitoring in the late 1980s and early 1990s.

Olanzapine, a second-generation (atypical) antipsychotic, primarily targets the dopamine and serotonin receptors to exert its effects. It acts as an antagonist on the D2 dopamine receptors found in the mesolimbic pathway, which prevents dopamine from interacting with the post-synaptic receptors. In terms of structure and pharmacological properties, olanzapine closely resembles clozapine. However, it distinguishes itself by the slower rate at which it dissociates from D2 receptors compared to clozapine [63]. This is due to its moderate affinity for these receptors. Both medications achieve their therapeutic effectiveness when the occupancy of D2 receptors reaches 65% [64]. Developed as a derivative of thienobenzodiazepine, olanzapine aims to find a substitute for clozapine without the need for frequent blood tests to monitor blood-related side effects.

The exact mechanism by which it treats schizophrenia, similar to other drugs, is not fully understood. However, it is believed that olanzapine’s effectiveness in controlling schizophrenia stems from its ability to inhibit both dopamine and serotonin type 2 (5HT2) receptors. Similarly, the precise process through which olanzapine aids in treating acute manic or mixed episodes in bipolar I disorder remains unclear.

The therapeutic benefits and side effects of olanzapine can be attributed to its action on receptors beyond dopamine and 5HT2. The drug’s counteraction of muscarinic M1-5 receptors may explain its anticholinergic-like effects. Similarly, the drowsiness associated with olanzapine could be due to its inhibition of histamine H1 receptors. Additionally, the orthostatic hypotension observed in patients taking olanzapine can be explained by its blockade of adrenergic α1 receptors [65].

## 5. Chronopharmacology in Psychiatric Diseases

Many medications are used every day in medical settings, often administered in the evening after assessing patients or as part of nightly routines. This scheduling is not based on chronotherapy, which is the strategy of timing medication administration for maximum effectiveness, but rather on the organization of clinical operations. A notable exception is statins, which are prescribed for evening use to reduce cholesterol production. Cholesterol synthesis follows a circadian rhythm, occurring mainly overnight (between midnight and 6:00 a.m.). This alignment of lipid-lowering treatment with the body’s natural cycles, a concept known as chronotherapy, suggests that the timing of medication administration can affect its effectiveness [66].

Another example is provided by the treatment of asthmatic patients with ampicillin, theophylline, and acetaminophen. The drugs were administered either before breakfast or at bedtime, and serum levels of these drugs were measured. The findings indicate that administering the medications at bedtime results in higher serum levels, aligning with the natural cycle of breathing difficulties [67,68]. Likewise, the effectiveness of TNP-470 in combating tumors is significantly higher in mice that received the drug during the early light phase compared to those injected during the early dark phase [69]. Similarly, the efficacy of SU1498, an inhibitor of VEGFR-2 tyrosine kinase that prevents the formation of new blood vessels, is greater when administered in the early light phase than in the early dark phase [70].

Arterial blood pressure serves as a crucial indicator of health, directly influencing the risk of organ damage and cardiovascular events. It is advisable to administer beta-blockers in the morning to reduce the activity of beta-1 and beta-2 adrenoreceptors. For individuals with high blood pressure who also suffer from increased heart rates, vascular α1- and α2-adrenergic receptors are noted to be responsible for the constriction of peripheral blood vessels. The concept of chronotherapy, adjusting treatment timing to the body’s rhythms, has been suggested for ensuring more effective management [71]. Research indicates that human catecholamine levels are lower at night and higher during the day [69], whereas rodents show the reverse pattern, aligning with their nocturnal nature [72,73]. This observation is critical, as many findings from animal studies on circadian rhythms and mental health have been directly applied to human medicine without considering these differences.

Growing interest is being directed towards understanding how the circadian system impacts the development and course of psychiatric disorders [74,75]. Nonetheless, the link between biomarkers of circadian rhythm and depression remains less clearly defined compared to their established connections with mania and schizophrenia [76].

**Figure 3:**
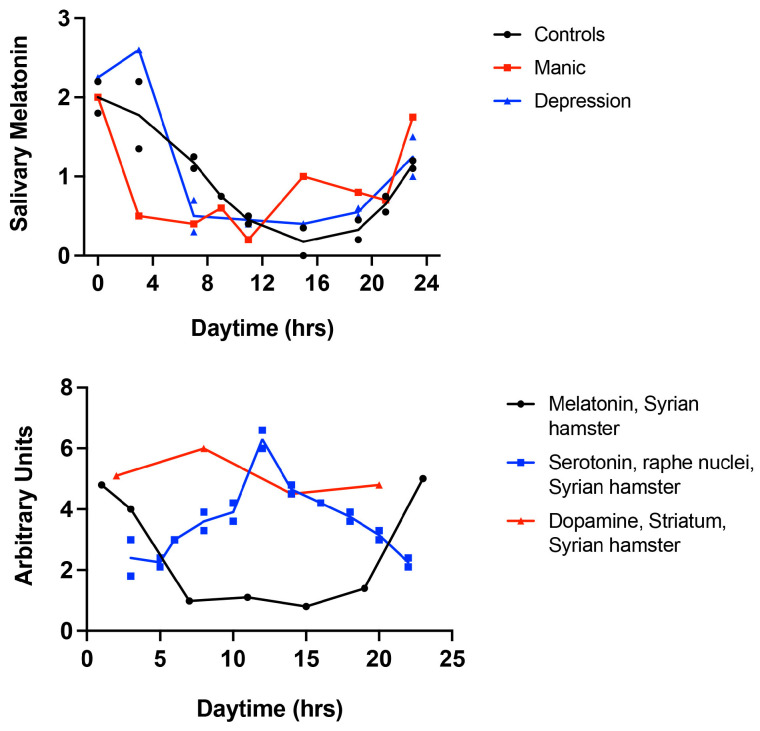
Circadian rhythmicity of melatonin, dopamine, and serotonin levels in mental disorders. (**Upper panel**): Circadian variations in salivary melatonin levels in controls, manic patients, and patients with major depression. (**Lower panel**): Levels of salivary melatonin, dopamine, and serotonin in the raphe nuclei of the Syrian hamster. The figure represents weighted median values based on information available in cited publications [49,78,79,80,81,82]. These values are presented in arbitrary units due to the use of different units for measuring neurotransmitter levels. The differences in neurotransmitter levels at various time points were determined by fold changes relative to time point zero. Please note the contrasting circadian rhythms of melatonin and serotonin. Melatonin data are derived from human subjects. For brain tissue analysis, we relied on studies of the Syrian hamster, whose circadian rhythms closely resemble those of humans.

Serotonin and melatonin display opposite patterns throughout the circadian cycle: serotonin peaks during the day, while melatonin levels increase at night [77]. As darkness falls, sympathetic fibers release noradrenaline, which triggers the release of serotonin stored within cells of the pineal gland and initiates melatonin synthesis, paving the way for the onset of sleep later on. Indeed, patients with cervical cord lesions leading to preganglionic sympathectomy, as well as diabetics suffering from clinical autonomic neuropathy, do not exhibit the typical daily rhythm of melatonin. This finding emphasizes the need to factor in circadian rhythms when devising treatment strategies for disorders such as mania and schizophrenia. It stresses the importance of taking into account the daily variations in cortisol, dopamine, and serotonin levels. Additionally, considering the activity of genes including *Bmal1*, *Clock*, and *Per1* in epithelial buccal cells, as well as changes in melatonin levels, is crucial (Figure 3).

The functional pairing of MT2 melatonin receptors with 5-HT2C receptors into active heteromers has garnered attention. This is due to the combined effects of melatonin agonism and 5-HT2C antagonism exhibited by agomelatine, a synthetic melatonergic agonist used to treat depression and generalized anxiety disorder [83,84]. Agomelatine is rapidly absorbed, undergoes swift metabolism in the liver, and is quickly eliminated with a half-life of 2.3 h [85].

Agomelatine exibits a unique chronobiological action that influences various aspects of the sleep–wake cycle. Thus, at night, agomelatine enhances sleep primarily through its melatonergic properties, while during the day, the drug promotes alertness by blocking the 5-HT2c receptors more strongly than it activates the melatonergic receptors [85,86]. Ramelteon is another synthetic melatonergic agonist targeting MT1 and MT2 receptors. It was the first melatonergic drug to receive US FDA approval in 2005 for its use as a sleep-inducing agent [87]. It is typically taken orally in the evening. Ramelteon is quickly absorbed, achieving peak plasma concentrations in under an hour. It has a short half-life, ranging from 0.83 to 1.90 h. However, its half-life exceeds that of melatonin [88].

As research continues to unveil the complex interplay between biological rhythms and disease processes, chronotherapy presents a promising opportunity for personalized medicine, optimizing treatment outcomes across a range of conditions. For instance, in cases of hypertension and asthma, administering medications at specific times can improve management and reduce exacerbations. Additionally, in cancer treatment, timing chemotherapy to align with the patient’s biological clock may enhance tumor sensitivity and reduce toxicity [89,90,91,92,93,94,95,96,97,98] (Table 1).

The potential benefits of evening dosing, such as reduced sedation and akathisia with medications like lurasidone, an antipsychotic drug used to treat schizophrenia, are significant [99,101]. However, the potential drawbacks—particularly regarding weight gain and the metabolic impact associated with evening dosing of medications like risperidone and olanzapine—cannot be overlooked, underscoring the importance of individualized patient care and the ongoing need for research to clarify these clinical uncertainties [100,102].

## 6. Circadian Changes in Pharmacokinetics and Pharmacodynamics

The central circadian clock is present not only in the brain but also in various peripheral tissues and organs. These are known as ‘peripheral clocks’ or ‘slave clocks’. These peripheral clocks synchronize with the central clock through mechanisms involving both nervous and hormonal signals. Acting as the primary rhythm source, the circadian clock regulates the 24 h cycles that affect drug effectiveness and toxicity. It is understood that the effectiveness levels and toxicities of many drugs vary according to circadian rhythms, depending on the time of administration, with differences in impact that can be up to tenfold.

Detailed examinations of key metabolic organs, such as the liver and kidney, which play a significant role in drug metabolism, underscore the importance of timing at both the proteomic and metabolomic levels [103,104].

Numerous medications, such as indomethacin, cyclosporine, nifedipine, valproic acid, theophylline, and digoxin, have shown variations that depend on the time of day in absorption and pharmacokinetics in both animals and humans [104,105]. The enzymes and transport mechanisms within the liver operate on a circadian rhythm, influenced by transcription factors that are regulated by the internal biological clock, coordinating the detoxification of foreign substances efficiently. Specifically, intestinal transporters like Slc15a1/PepT1 and Slc22a4/Octn1, crucial for the absorption of drugs, exhibit a circadian rhythm in their expression levels, indicating how these levels vary throughout the day [106,107,108].

Variations in the circadian-dependent processing of drugs have also been extensively reported for the liver’s processing of drugs through glucuronidation, as well as the metabolism of substances like steroids, aminopyrine, hexobarbital, imipramine, and benzphetamine [106]. Furthermore, several genes involved in drug metabolism in humans, including *CYP2D6*, *CYP2E1*, and *CYP3A4*, have been identified as potential circadian genes. However, further human studies are required for confirmation [106,107,108,109,110,111,112].

It is also well documented that kidney function undergoes circadian rhythms [113]. For instance, in humans, various substances that are excreted in urine display daily fluctuations in their pharmacokinetic elimination parameters. Thus, the anticancer precursors of 5-fluorouracil, namely tegafur and capecitabine, exhibit increased total oral clearance at 11 p.m. Additionally, the projected average clearance of 5-fluorouracil reaches its peak between 8 and 10 a.m. [114].

### 6.1. Pharmacokinetics and Pharmacodynamics in Psychiatric Diseases

The pharmacokinetic and pharmacodynamic profiles of medications commonly used in psychiatric settings are under-researched. Haloperidol, a first-generation antipsychotic, primarily targets D2 receptors, which explains the limited effectiveness of traditional antipsychotics in treating mood disorders. The way haloperidol is processed in the body varies according to individual factors such as sex, age, body weight, and ethnicity. In healthy individuals, the elimination half-life of Haloperidol after a single oral dose is reported to be between 14.5 and 36.7 h, or up to 1.5 days. However, with long-term use, the half-life can extend to as much as 21 days. This indicates that the elimination half-life of Haloperidol is significantly longer with chronic use compared to its half-life after a single dose [115]. However, the long half-life of haloperidol, well beyond 24 h, makes its interpretation within the context of chronotherapy, which considers the circadian timescale, challenging. This reflects the inherent limitations and boundaries of psychiatric chronopharmacology [116].

#### 6.1.1. *Risperidone* Pharmacodynamics

The daily dose of risperidone, administered twice daily, ranges from 0.5 to 8 mg. The exact time of administration is not known but most likely depends on the clinic’s routine. Risperidone is orally bioavailable, with an peak of 70% [117]. The peak concentration of risperidone is achieved approximately 1 h after administration. In contrast, the peak time for 9-hydroxyrisperidone varies based on the individual’s CYP2D6 metabolic profile: it occurs 3 h post-administration in individuals with normal metabolism and 13 h in poor metabolizers [118].

In serum, risperidone and 9-hydroxyrisperidone are bound to plasma proteins, specifically albumin and α1-acid glycoprotein. The elimination of risperidone and its metabolites primarily occurs through the kidneys [119]. The pharmacokinetic profiles of risperidone and 9-hydroxyrisperidone, for both single and repeated doses, show similarity between normal and poor metabolizers, with an average half-life of about 20 h [118].

#### 6.1.2. Olanzapine Pharmacodynamics

Studies have demonstrated that daily doses of 12 mg or more can block at least 65% of D2 receptors in the striatum, and doses exceeding 20 mg can achieve receptor occupancy rates above 80% [120]. The pharmacological effects of olanzapine are primarily associated with its action in the mesolimbic and mesocortical areas of the brain [121]. Research on olanzapine’s pharmacokinetics reveals that it follows linear kinetics across the doses used in clinical practice. Its elimination half-life varies from 21 to 54 h, with its apparent plasma clearance ranging between 12 and 47 L/hr. The administration of olanzapine once daily results in stable blood levels within approximately one week, with concentrations being about twice as high as those observed after a single dose.

Factors such as smoking status, gender, and age can lead to variations in plasma concentrations, half-life, and clearance rates of olanzapine among patients. The drug is widely distributed in the body, having a distribution volume of around 1000 L, and is 93% plasma protein-bound across a concentration range of 7 to 1100 ng/mL, mainly to albumin and α1-acid glycoprotein. Variations in the plasma concentration, half-life, and clearance rate of olanzapine among patients can be influenced by factors such as smoking status, gender, and age [122,123] (Table 2).

## 7. Towards a Chronotherapy for Psychiatric Patients

In the context of antidepressive treatment, “chronotherapy” may signify non-pharmacological, non-invasive strategies aimed at altering sleep patterns. This includes practices such as sleep deprivation, adjusting sleep phases, and utilizing bright light therapy. These methods are employed to mitigate symptoms in conditions thought to stem from disruptions in circadian rhythms, such as depression, as previously suggested [124,125]. For example, traditional antidepressants often take 2 to 8 weeks before showing effects. As such, there has been ongoing effort for years to find a quick and lasting treatment for depression in bipolar disorder (BPD). The fastest documented treatment so far is sleep deprivation (SD), which can improve symptoms in 40% to 60% of patients within 24 to 48 h. This suggests that treatments taking into account circadian rhythmicity are extremely beneficial to patients in terms of rapid and sustained response, allowing for lower drug doses [126].

A small-scale observational study highlighted significant improvements in depression among patients treated with lithium or sertraline who also received adjunctive non-invasive chronotherapy. This regimen combined sleep deprivation, two hours of bright light therapy (starting the morning after the sleep-deprived night) for three days, and sleep phase advance (beginning the evening after sleep deprivation), as reported by Wu and colleagues [126].

Two systematic analyses have explored the efficacy and tolerability of major chronotherapeutic interventions in treating bipolar disorder, including their integration with routine care for bipolar depression. These analyses suggest that although the effectiveness of chronotherapeutic methods may vary due to factors such as study scale, quality, and strength of evidence, ‘Triple Chronotherapy’ has shown potential as a beneficial supplement to standard antidepressant therapies [127,128].

Recent research has identified deficiencies in key circadian rhythm genes, including *CLOCK*, PER, CRY, and *BMAL1*, in bipolar disorder, depression, and autism spectrum disorder. Similar gene deficiencies are noted in schizophrenia (*CLOCK*, PER, CRY), anxiety disorder (*CRY*), and attention deficit hyperactivity disorder (ADHD) (*CLOCK*) [129].

In the case of antipsychotic treatments that utilize dopamine and serotonin receptor antagonists, the optimal timing for administration should be established based on circadian variation in biomarker profiles. These biomarkers include cortisol, melatonin, dopamine, serotonin, and key clock genes such as *CLOCK* and BMAL1, aiming to achieve maximum efficacy with minimal toxicity and side effects. Thus, in a study conducted on manic patients, melatonin levels were increased during the day and correlated with shifts in the expression of *PER1* and NR1D1 mRNAs in the buccal cells of patients diagnosed with mania [49].

Mood disorders frequently correlate with disturbances in sleep cycles [130]. The SCN nucleus receives inputs from a serotonergic network originating in the raphe nuclei and from two light-sensitive sources. Light directly influences melatonin synthesis through the SCN’s response to light exposure, which then modulates the activity of the pineal gland. The raphe nuclei contribute by regulating serotonin levels, which are crucial for melatonin production. Thus, the interplay between light exposure, SCN signaling, raphe nuclei activity, and pineal gland function orchestrates the synthesis and timing of melatonin release. This serotonergic network regulates circadian responses in the SCN to various stimuli. In addition, although there are no direct connections from the nigrostriatal system to the SCN, dopamine influences circadian rhythms indirectly through serotonin pathways, impacting both physical and mental health. Atypical antipsychotics exert their action primarily as dopamine antagonists and also modulate serotonin, norepinephrine, and/or histamine neurotransmission. During night-time, when the SCN’s inhibitory influence is lowered, the activity of the raphe nuclei decreases, leading to reduced serotonin release. We hypothesize that administering atypical antipsychotics in the late evening favors the conversion of serotonin to melatonin in the pineal gland, thereby improving sleep quality in psychiatric conditions such as schizophrenia.

Given the contrasting circadian rhythms of melatonin and serotonin (see Figure 3), administering antipsychotic drugs, including risperidone, olanzapine, quetiapine, ziprasidone, aripiprazole, brexpiprazole, paliperidone, asenapine, lurasidone, iloperidone, cariprazine, and clozapine, around 9 p.m. would be optimal. Indeed, on occasion, some psychiatrists prefer to prescribe olanzapine to be taken at night based on their experience [131].

When determining optimal dosing, it is crucial to consider the peripheral half-life. Equally important, however, is the CNS half-life, as the CNS is the primary target in psychiatric treatments. For many psychotropic medications, the CNS half-life exceeds 24 h [101]. This extended CNS half-life supports the rationale for once-daily dosing, especially in the evening, to maximize therapeutic benefits while reducing side effects and enhancing patient adherence. Administering medications multiple times a day can increase the likelihood of adverse effects. Adopting a patient-centered approach allows for the customization of therapeutic strategies to achieve the best possible outcomes for each individual.

### 7.1. Steps Towards a Chronotherapy for Psychiatric Patients

#### 7.1.1. Measuring the Activity Individually by Using Actiwatches

Assessing patient activity levels with Actiwatches is a common practice. These wrist-worn accelerometers track physical movement and are frequently employed to evaluate circadian rhythms and disrupted sleep patterns.

#### 7.1.2. Measuring Biomarkers of the Circadian Rhythm

In usual clinical practice, drugs are administered once per day, typically either in the morning or in the evening. However, given that drug absorption, biodistribution, effects on target organs, half-life, metabolism, and elimination are all influenced by the circadian rhythms of our body’s physiology, a question arises about the optimal time of administration required in order to achieve maximum efficacy in treating psychiatric diseases. These diseases are dependent on the circadian rhythms of our body’s physiology, including day–night sleep cycles.

Hence, initiating chronotherapy begins with the crucial step of precisely determining the patient’s circadian rhythm biomarkers in saliva, including cortisol, melatonin, and key genes that regulate the circadian clock, such as *CLOCK*, *BMAL1*, *PER1*, *PER2*, and *PER3*-in buccal cells. Serum concentrations of dopamine and serotonin are less relevant because what is important is their variations in the thalamus and raphe nuclei, respectively. Here, we must rely on studies in animal models, especially the Syrian hamster, which has a circadian rhythmicity resembling that of humans. It is doubtful that postmortem analysis of human brain tissue would be of any help.

Despite the substantial benefits of circadian medicine, its progress has been hindered by a lack of non-invasive, complex devices for analyzing data and automatically suggesting the best times for treatment. However, recently, a device named TimeTeller has been developed as a non-invasive tool that combines molecular and digital technologies to evaluate circadian rhythms and predict daily behaviors, including the best times for medication administration. This breakthrough seeks to broaden the use of circadian medicine in various settings. Given the range of health aspects, both recognized and yet to be identified, that are influenced by patient circadian rhythms, the introduction of this new biomarker significantly enhances personalized healthcare. It utilizes comprehensive health information from the lifestyle, medical care, and research fields [132]. Importantly, applying this method to patients with bipolar disorder has successfully regulated their irregular circadian patterns of cortisol and melatonin [43,49,78].

## 8. Methodology

Many medications are administered daily in medical settings, often in the evening, after patient assessments, or as part of nightly routines. In this study, we examine whether this scheduling is based not on chronotherapy—a strategy that involves timing medication administration for maximum effectiveness—but rather on the organization of clinical operations. To achieve this objective, we conducted a comprehensive search of the relevant scientific literature using two major databases: PubMed and Web of Science. The search terms that we employed included ‘circadian rhythmicity’, ‘cortisol’, ‘melatonin’, ‘serotonin’, ‘dopamine’, ‘clock genes’, ‘dopamine receptor’, ‘hypothalamus-pituitary-adrenal (HPA) axis’, ‘5-hydroxytryptamine (serotonin)’, ‘5-hydroxytryptamine receptor type 2’, ‘major depressive disorder’, ‘sleep deprivation’, ‘SCN nuclei’, ‘single-nucleotide polymorphisms’, ‘mental health’, ‘psychiatric disorders’, ‘bipolar disorders’, ‘schizophrenia’, ‘attention-deficit/hyperactivity disorder’, ‘time of administration’, and ‘chronopharmacology’. To ensure a comprehensive approach, we included a wide array of both qualitative and quantitative studies, comprising primary research articles and review papers. Our literature search encompassed studies published up to the date of this review, ensuring the inclusion of the most recent research findings in the field. Given the flexibility that a review article affords, we employed thematic analysis to synthesize the findings, identifying patterns and common themes across the selected literature

## 9. Conclusions

(1) In acute settings where high doses are typically administered, the timing of drug administration is less critical. However, as the dosage decreases, timing in relation to the body’s circadian rhythms becomes increasingly important. Properly aligning drug administration with these natural rhythms can reduce the burden on the liver and kidneys, significantly enhancing the drug’s effectiveness at lower doses.

(2) The efficacy of post-acute-phase antipsychotic treatment could be improved by considering the biomarkers of circadian rhythmicity, such as cortisol and melatonin levels, along with the buccal gene expression of *BMAL1*, *CLOCK*, and *PER1*, *PER2*, *PER3* transcripts. These can serve both as indicators of efficacy and as guides on how to make timely adjustments to and optimize drug therapy. However, it should be noted that recommendations regarding drug approval, safety, prescription, and management are issued by regulatory bodies in the European Union. Each EU Member State also has its own rules for prescription medications.

(3) Given the opposite circadian profiles of melatonin and serotonin during the sleep–wake cycle, the best time to administer the second-generation antipsychotic drugs would be around 9 p.m.

(4) The question remains whether psychiatric disorders are initially caused by genetic predispositions that disrupt circadian rhythms, or if these disruptions are a result of the disorder. Considering the circadian system’s bidirectional relationship with the neural pathways that regulate behavior, it is plausible that both elements play a role in the development of psychiatric conditions.

## Figures and Tables

**Table 1:** The optimal time for the administration for several drugs.

Drug	Effect	Optimal Time of Administration	Reference
Statins	LDL-C and TC lowering	Evening	[89]
Aspirin	Anti-thrombotic	Evening	[90]
Insulin glargine		Morning	[91]
Antihypertensive	Lower blood pressure	Bedtime	[92]
Capecitabine	Anti-cancer	9 a.m. and 0 a.m.	[93]
Oxaliplatin		4 p.m.	[94]
Doxorubicin	Anti-ovarian cancer	6 a.m.	[94]
Cisplatin	Anti-ovarian cancer	4 p.m.–20 p.m.	[94]
Immune checkpoint inhibitors	Management of advanced melanoma	4:30 p.m.	[95]
Mequitazine; Histamine H1 receptor antagonist	Allergies and rhinitis	7 p.m.	[96]
Theophylline	Bronchial asthma	Tablets every six hours	[97]
Gabapentin	Management of neuropathic pain	8 p.m.	[98]
Lurasidone	Anti-psychotic, schizophrenia	Evening	[99]
Risperidone	Anti-psychotic, schizophrenia	Evening	[100]

**Table 2:** The key points regarding the pharmacokinetics, pharmacodynamics, and influencing factors for haloperidol, risperidone, and olanzapine in psychiatric treatment.

Medication	Target/Action	Pharmacokinetics	Pharmacodynamics	Factors Influencing
Haloperidol	Primarily targets D2 receptors	-Elimination half-life after single oral dose: 14.5–36.7 hrs -Chronic use. Up to 21 days	Challenges with chronotherapy due to long half-life	Sex, age, body weight, ethnicity
Risperidone	5-HT25-HT2A receptors	-Daily dose: 2–8 mg, twice daily -Oral bioavailability: 70% -Peak concentration: 1 hr after administration -Elimination: primarily through kidneys -Half-life: 20 h	-Peaktime:3 h (normal metabolizers), 13 h (poor metabolizers)	-CYP2D6 metabolic profile
Olanzapine	Blocks D2 receptors in the striatum	-Elimination half-life: 21–54 h -Plasma clearance: 12–47 L/hr -Distribution volume:~1000 L -93% plasma protein bound	-Blocks ~65% of D2 receptors at 12 mg -Blocks >80% of D2 receptors at 20 mg -moderate affinity at M3), and serotonin receptors (5-HT2A/C, and 5-HT3/6 receptors)	-Smoking status, gender, age

## Data Availability

The data that support the findings of this study are available from the corresponding author upon reasonable request.
